# Anti-infectious and anti-inflammatory effects of peptide fragments sequentially derived from the antimicrobial peptide centrocin 1 isolated from the green sea urchin, *Strongylocentrotus droebachiensis*

**DOI:** 10.1186/2191-0855-2-67

**Published:** 2012-12-13

**Authors:** Camilla Björn, Joakim Håkansson, Emma Myhrman, Veronika Sjöstrand, Tor Haug, Kerstin Lindgren, Hans-Matti Blencke, Klara Stensvåg, Margit Mahlapuu

**Affiliations:** 1 Pergamum AB, Arvid Wallgrens Backe 20, 413 46, Gothenburg, Sweden; 2 Present address: SP Technical Research Institute of Sweden, Medical Device Technology, Brinellgatan 4, 504 62, Borås, Sweden; 3 Norwegian College of Fishery Science, Faculty of Biosciences, Fisheries and Economics, University of Tromsø, Tromsø N-9037, Norway

**Keywords:** Antibacterial peptide, Wound infection, Antibiotic resistance, MRSA

## Abstract

Bacterial resistance against antibiotic treatment has become a major threat to public health. Antimicrobial peptides (AMPs) have emerged as promising alternative agents for treatment of infectious diseases. This study characterizes novel synthetic peptides sequentially derived from the AMP centrocin 1, isolated from the green sea urchin, for their applicability as anti-infective agents.

The microbicidal effect of centrocin 1 heavy chain (CEN1 HC-Br), its debrominated analogue (CEN1 HC), the C-terminal truncated variants of both peptides, *i.e.* CEN1 HC-Br (1–20) and CEN1 HC (1–20), as well as the cysteine to serine substituted equivalent CEN1 HC (Ser) was evaluated using minimal microbicidal concentration assay. The anti-inflammatory properties were assessed by measuring the inhibition of secretion of pro-inflammatory cytokines. All the peptides tested exhibited marked microbicidal and anti-inflammatory properties. No difference in efficacy was seen comparing CEN1 HC-Br and CEN1 HC, while the brominated variant had higher cytotoxicity. C-terminal truncation of both peptides reduced salt-tolerability of the microbicidal effect as well as anti-inflammatory actions. Also, serine substitution of cysteine residue decreased the microbicidal effect. Thus, from the peptide variants tested, CEN1 HC showed the best efficacy and safety profile. Further, CEN1 HC significantly reduced bacterial counts in two different animal models of infected wounds, while *Staphylococcus aureus* and methicillin-resistant *S. aureus* (MRSA) failed to develop resistance against this peptide under continued selection pressure. In summary, CEN1 HC appears a promising new antimicrobial agent, and clinical studies are warranted to evaluate the applicability of this AMP for local treatment of infections in man.

## Introduction

Infectious diseases are among the leading causes of death worldwide. According to the World Health Organization, acute infectious diseases account for 25% of deaths globally and for 45% in developing countries, killing about 15 million people per year (WHO 2004). Several orders of magnitude higher is the number of people who survive the initial infection but suffer from conditions where microbes cause a chronic inflammation (Schmidt 2004). The treatment of infections has recently been complicated due to emerging increase of bacterial resistance towards conventional antibiotics. The occurrence of MRSA in hospital environments has been reported as a significant problem since its recognition over 50 years ago (Jevons 1961). In recent years, the emergence of community-acquired strains has rapidly grown, as in some settings, community-acquired MRSA represents greater than 50% of the total *S. aureus* infections (Giordano et al. 2007). In addition, enterococci, leading to surgical wound and urinary tract infections, are becoming intrinsically resistant to many antibiotics. Also, multi-drug resistant strains of *Pseudomonas aeruginosa* are associated with severe adverse clinical outcomes (Jones et al. 2005; Moet et al. 2007). Resistance development is not confined to antibiotics only. Silver products have been considered to carry a low risk of resistance development, however burn wound studies have demonstrated that bacteria, in particular *P. aeruginosa*, may become resistant to silver sulfadiazine and silver nitrate (Vermeulen et al. 2007).

AMPs are receiving increased attention as potential therapeutic candidates in infectious disease treatment. AMPs are gene-encoded, ribosomally synthesised peptides which are widespread in nature, and appear to be important defence molecules in all eukaryotic phyla. Most naturally occurring AMPs carry a net positive charge (*i.e.* cationic) and are composed of 12–50 residues where close to 50% of them are hydrophobic (Hancock and Diamond 2000; Teixeira et al. 2012). In membrane-like environments AMPs tend to form amphipathic structures, *i.e.* structures with separate hydrophobic and hydrophilic domains. The positive charge of AMPs presumably enables interactions with the negatively charged outer leaflet of bacterial membranes and/or bacterial cell wall components, whereas their amphipathic character causes membrane permeabilization (Devine and Hancock 2002; Teixeira et al. 2012). Notably, although AMPs are widely distributed in nature and bacteria have been exposed to these molecules for millions of years, widespread resistance has not been reported (Fjell et al. 2012). Many AMPs have also shown to possess favourable activities other than bactericidal effect, such as anti-inflammatory properties (Auvynet and Rosenstein 2009; Bowdish et al. 2005; Scott and Hancock 2000). Based on these properties, AMPs have emerged as novel promising agents for both topical and systemic treatment of infectious diseases (Schuerholz et al. 2012; Yount and Yeaman 2012).

Invertebrates are proving to be a rich source of AMPs, possibly because of their more pronounced reliance on innate immune functions in their defence against microbial pathogens compared to vertebrates. Many of the peptides isolated from marine species are structurally unique, display significant biological activities and are therefore expected to be useful drug leads (Sperstad et al. 2011). In a previous study, we detected broad-spectrum antibacterial activity in coelomocyte (blood cell) extracts of the green sea urchin, *S. droebachiensis* (Haug et al. 2002). Subsequently, an AMP named centrocin 1 was isolated and characterized. Centrocin 1 is a heterodimer formed by monomers of 30 (heavy chain) and 12 amino acid residues (light chain) connected by a single disulphide bridge (Li et al. 2010). Synthesis and subsequent antimicrobial testing of the different monomers showed that the cationic heavy chain is equally active as the original dimeric peptide. On the other hand, the neutral light chain showed no antimicrobial activity (Li et al. 2010). Helical wheel projection of the heavy chain indicates that this sequence has an amphipathic structure, a feature common for many membrane-active AMPs. Interestingly, centrocin 1 heavy chain contains a brominated tryptophan on position two (Li et al. 2010) with unknown function.

In the present study, centrocin 1 heavy chain (CEN1 HC-Br) and derivatives thereof, were synthesised and screened for *in vitro* bactericidal activity against selected microbial strains in four different assay media. The peptides were also evaluated for *in vitro* anti-inflammatory properties as measured by inhibition of secretion of tumor necrosis factor alpha (TNF-α) and interleukin-6 (IL-6) from lipopolysaccharide (LPS) stimulated macrophages derived from the monocytic cell line THP-1, and for their cytotoxicity to the same cells. The debrominated analogue of centrocin 1 heavy chain, CEN1 HC, was evaluated for its ability to reduce bacterial counts in two different infected wound models, an *in vivo* excision wound model in rats, and an *ex vivo* pig skin model. In addition, resistance development against CEN1 HC was assessed in the bacterial strains *S. aureus* and MRSA.

## Materials and methods

### Peptides and antibiotics

The heavy chain of centrocin 1 (CEN1 HC-Br), the C-terminal truncated peptide (CEN1 HC-Br (1–20)) and its debrominated equivalent (CEN1 HC (1–20)) were purchased from BIOMOL International LP (Exeter, UK). The non-halogenated heavy chain CEN1 HC and an analogue where the free cysteine in position 25 is exchanged with serine, CEN1 HC (Ser), were purchased from Biopeptide Co., Inc. (San Diego, CA). The peptides (Table 1) were all synthesised using Fmoc solid phase technology, and the peptide content and purity were determined by high performance liquid chromatography (HPLC) and mass spectrometry (MS) analysis. The brominated peptides were all synthesised with 5-Bromo-D/L-tryptophan in position 2. Mupirocin (Bactroban, 2% ointment, GlaxoSmithKline, Brentford, UK) and fusidic acid (Fucidin, 2% cream, LEO Pharma, Malmö, Sweden) were used as comparators in assessment of *in vitro* microbicidal effect. In the *in vitro* resistance assay, mupirocin (Applichem, Darmstadt, Germany) in powder form was used as comparator.

**Table 1:** Amino acid sequence of centrocin 1 heavy chain (CEN1 HC-Br) and its synthetic fragments and analogues

**Peptide**	**Sequence**	**Net charge at pH = 7.0**	**Ratio hydrophobic/total residues**
CEN1 HC-Br	GW(Br)FKKTFHKVSHAVKSGIHAGQRGCSALGF	+ 5.2	53%
CEN1 HC	GWFKKTFHKVSHAVKSGIHAGQRGCSALGF	+ 5.2	53%
CEN1 HC-Br (1–20)	GW(Br)FKKTFHKVSHAVKSGIHA	+ 4.3	60%
CEN1 HC (1–20)	GWFKKTFHKVSHAVKSGIHA	+ 4.3	60%
CEN1 HC (Ser)	GWFKKTFHKVSHAVKSGIHAGQRGSSALGF	+ 5.3	50%

### *In vitro* microbicidal effect

All the synthesised peptides were tested for their *in vitro* microbicidal activity against *S. aureus* (American Type Culture Collection (ATCC) 12600) and *P. aeruginosa* (ATCC 15442) using a minimal microbicidal concentration (MMC) assay as previously described (Haversen et al. 2010). Bacteria were cultured in 3.7% brain heart infusion (BHI) broth (Difco, BD Diagnostics, Franklin Lakes, NJ) on a shaker at 250 rpm overnight at 37°C. The culture was diluted 1:10 in fresh 3.7% BHI and incubated for two additional hours to reach log-phase growth. The bacteria were pelleted by centrifugation at 900 × g and suspended in 0.037% BHI to a concentration of 10^7^ CFU/mL, estimated by measuring optical density at 600 nm (OD_600_). To investigate the influence of physiological conditions on microbicidal effect of the peptides, four different assay media were used: 0.037% BHI, 0.037% BHI supplemented with 85 or 150 mM sodium chloride (NaCl), and 50% heat inactivated simulated wound fluid (h.i. SWF) composed of a 50:50 mixture of 0.1% peptone (Oxoid, Basingstoke, UK) in 150 mM NaCl and fetal bovine serum (FBS; PAA Laboratories GmbH, Pasching, Austria), diluted two times in ultra pure water. The peptides were serially diluted by twofold steps from 200 to 1.56 mg/L in the assay media used, and 100 μL of the peptide solutions were mixed with 5 μL bacterial suspensions in a 96 well plate (Nunc, Roskilde, Denmark) and incubated at 37°C for two hours. An aliquot of 5 μL from each well was aspirated and added as drops onto blood agar plates (Columbia agar; Oxoid, Basingstoke, UK) supplemented with 5% defibrinated horse blood (Swedish National Veterinary Institute (SVA), Uppsala, Sweden), and the plates were incubated overnight at 37°C. All samples were processed as duplicates. The minimal test compound concentration causing ≥99% reduction of bacteria was defined as the MMC_99_. The concentration of the bacterial suspension used in the assay was confirmed by viable count estimation on blood agar plates (Oxoid).

CEN1 HC was further screened for microbicidal activity against a panel of microbial strains using the MMC_99_ assay, as described above, except using only two different assay media; 0.037% BHI and 50% h.i. SWF. The following microbial strains were tested for sensitivity: *S. aureus* (ATCC 12600), MRSA (ATCC 33591), *Staphylococcus epidermidis* (ATCC 12228), *Streptococcus pyogenes* (ATCC 12344), *Propionibacterium acnes* (ATCC 6919) *Escherichia coli* (ATCC 11775), *P. aeruginosa* (ATCC 15442), *Klebsiella pneumoniae* (Culture collection, University of Gothenburg (CCUG) 59413, clinical isolate resistant to penicillins, cephalosporins, aztreonam and carbapenems, with the reference strain ATCC 13883), *Acinetobacter baumannii* (CCUG 58437, clinical isolate resistant to tobramycin, trimsulfa, ciprofloxacin, cefotaxim, ceftazidim, meropenem, pipera/tazobactam, with the reference strain ATCC 19606) and the yeast strain *Candida albicans* (ATCC 64549). Mupirocin (GlaxoSmithKline) and fusidic acid (LEO Pharma) were used as comparative control antibiotics.

### *In vitro* anti-inflammatory effect

The human monocytic cell line THP-1 (ATCC TIB-202) was cultured in RPMI 1640 medium (PAA Laboratories GmbH) supplemented with 10% FBS (PAA Laboratories GmbH), 1 mM sodium pyruvate (Sigma-Aldrich, St. Louis, MO), and 20 mM 4-(2-hydroxyethyl)-1-piperazineethanesulfonic acid (HEPES; PAA Laboratories GmbH). The cell density was adjusted to 10^6^ cells/mL and 100 μL of the cell suspension was added to each well of 96 well plates (Sarstedt, Nümbrecht, Germany). The cells were treated with 10 ng/mL phorbol 12-myristate 13-acetate (PMA; Sigma-Aldrich) to differentiate the monocytes into macrophage-like cells. After 48 h, the cells were stimulated by adding 0.1 ng/mL LPS into the medium specified above, except that 10% FBS was replaced with 5% h.i. FBS. The LPS concentration used was selected based on *in vitro* titration experiments to give a close to maximum release of cytokines into the cell culture medium over a period of six hours (data not shown). The indicated concentrations of the peptides were added 30 min after addition of LPS. After six hours of incubation, the plates were centrifuged for six minutes at 400 × g and the supernatants were collected and kept frozen at −20°C until analysed for TNF-α and IL-6 production. The cytokine levels were analysed using enzyme-linked immunosorbent assay (ELISA; R&D Systems, Minneapolis, MN).

### *In vitro* cytotoxic effect on THP-1 cells assessed by TACS MTT assay

THP-1 cells were cultured, differentiated, stimulated and the peptides were added as described above. Triton X-100 (ICN Biomedicals Inc., OH) was used as a positive control, and added at the same time as the peptides. After six hours of incubation, TACS MTT (3-(4, 5-Dimethylthiazol-2-yl)-2, 5-diphenyltetrazolium bromide) assay (R&D Systems) was performed according to the manufacturer’s instructions. In short, 10 μL MTT reagent was added to each well and the plates were incubated for two hours at 37°C. Subsequently, 100 μL detergent reagent (100 mg/L) was added and the plates were incubated in dark at room temperature overnight. The plates were shaken for 10 min at 300 rpm and the absorbance at 570 nm with the reference wavelength 650 nm was measured.

### *In vitro* resistance assay

*S. aureus* (ATCC 12600) and MRSA (ATCC 33591) were cultured according to the protocol described above and diluted to 10^8^ CFU/mL in 0.037% BHI as estimated by measuring OD_600_. The multistep resistance assay was performed as previously described (Kosowska-Shick et al. 2006). Peptide dilutions were prepared in twofold dilution steps in the concentration range from 0.75 mg/L to 96 mg/L in a volume of 1 mL using 0.37% BHI medium. The same peptide concentration range was used during all passages and new peptide dilutions were prepared each day from stock solutions. Mupirocin (Applichem), the active substance in the conventional topical antibiotic Bactroban, was used as a comparator. The antibiotic control was handled in the same way as the peptides except that the concentration range of mupirocin was 0.003 mg/L to 3 mg/L.

An aliquot of 10 μL (10^8^ CFU/mL) of the bacterial suspension was added to the peptide dilutions, to the positive antibiotic control, and to a negative control incubated without peptide, and the suspensions were incubated at 37°C for 24 h. Turbidity of the overnight cultures was determined by OD_600_ measurements. The OD_600_ of bacteria/peptide suspensions was calculated by subtracting the OD_600_ of the peptide mixed with medium from the OD_600_ measured on the overnight cultures. To create an optimal selection pressure, the overnight culture having the highest peptide concentration without marked reduction of bacterial viability (defined as OD_600_ value of >85% compared to the value measured for the negative control sample) was further passed.

The bacteria/peptide suspension to be further passed was diluted in 0.37% BHI to 10^8^ CFU/mL. A volume of 10 μL (10^8^ CFU/mL) from the selected inoculum was transferred to vials with 1 mL fresh peptide solutions diluted in 0.37% BHI. The suspensions were incubated at 37°C for 24 h. The same procedure was performed with the antibiotic control. The remaining bacterial suspensions were centrifuged at 900 × g for ten minutes, then resuspended in a 50:50 mixture of 3.7% BHI and 80% glycerol and immediately frozen at −80°C and kept frozen until analysis. The 24-h incubation and inoculation procedure described above was repeated for 14 days. MMC_99_ assay was performed, as described above, on the overnight cultures after 0, 1, 4, 7 and 14 passages to evaluate resistance induction.

### *In vivo* excision wound model in rats

The ability of CEN1 HC and CEN1 HC (Ser) to reduce bacterial counts was estimated in an excision wound model in rat designed based on previously published studies (Gisby and Bryant 2000; McRipley and Whitney 1976; Rittenhouse et al. 2006; Saymen et al. 1972). Briefly, female Sprague–Dawley rats (200–250 g, Charles River Laboratories, Sulzfeldt, Germany) were housed at the Laboratory of Experimental Biomedicine, Gothenburg, Sweden. They were kept in a 12-h light–dark cycle and were cared for in accordance with regulations for the protection of laboratory animals. All animal experiments were performed after prior approval from the local Ethics Committee for Animal Studies at the Administrative Court of Appeals in Gothenburg, Sweden. The rats were acclimatised in cages for a minimum of five days before surgery, with four rats in each cage. They had free access to water and pellets (Lab For, Lantmännen, Sweden). The rats were anaesthetised during the whole experiment and the anaesthesia was induced by an intra-peritoneal injection of a mixture of fentanyl (272 μg/kg; B. Braun Melsungen AG, Melsungen, Germany) and medetomidine hydrochloride (545 μg/kg; Domitor, Orion Pharma Animal Health AB, Sollentuna, Sweden). The backs of the rats were shaved and swabbed with 70% chlorhexidine alcohol (Fresenius Kabi AB, Uppsala, Sweden). Six 10 mm × 10 mm full thickness wounds were made, separated by the distance of 5 mm. Bacteria were prepared according to the same protocol as described above and diluted to 2 × 10^9^ CFU/mL. The wounds were seeded with 20 μL of either MRSA (ATCC 33591) or *P. aeruginosa* (ATCC 10145) for the treatment with CEN1 HC, and with 20 μL of MRSA (ATCC 33591) for the treatment with CEN1 HC (Ser). Two hours after infection, 100 μL of peptide in H_2_O (0.1–2 mg/mL) or placebo (H_2_O) was added to each wound. The treatment was randomised for all wounds. All animals were euthanized two hours after treatment by an overdose of pentobarbital sodium (Pentobarbital vet, APL, Stockholm, Sweden). To estimate bacterial counts, the whole wound area was dissected and transferred to a micro-centrifuge tube and placed on ice. A volume of 500 μL of Kligman buffer (0.1% Triton X-100 in 0.075 M phosphate buffer, pH 7.9) was added and the tube was vortexed for two minutes, followed by shaking for ten minutes at 1400 rpm. Each suspension was diluted in four tenfold serial steps in diluted Kligman buffer (0.05% Triton X-100 in 0.0375 M phosphate buffer, pH 7.9). Aliquots of 50 μL from each of the four dilutions were seeded on blood agar plates (Oxoid) and incubated at 37°C overnight. Plates containing 30–300 CFU were counted. All personnel involved in the surgery, harvesting the bacteria and counting the plates were blinded to the different treatments.

### *Ex vivo* model using pig skin

The ability of CEN1 HC and CEN1 HC (Ser) to reduce bacterial counts was estimated in an *ex vivo* wound model using pig skin as previously described (Schmidtchen et al. 2009). The pigs used in the study were a mixed breed of Yorkshire, Hampshire and Swedish Pigham. The pig was shaved after euthanization and skin from the back was removed, packed in plastic foil and frozen at −20°C for storage. Before the experiment, the skin was taken out from the freezer and the subcutaneous fat was removed with a scalpel. The skin was put in a petri-dish with Kleenex paper tissues (Kimberly Clark GmbH, Koblenz, Germany) in the bottom, wetted with sterile H_2_O, and cleaned with 70% ethanol. Punch biopsies were made, approximately 0.5–1 mm in thickness and 3 mm in diameter. The top cylinder of a cut 1.5 mL micro-centrifuge tube (diameter approximately 9 mm) was glued around each punch wound with ethyl cyanoacrylate glue (Loctite super glue gel, Henkel Norden, Stockholm, Sweden). The area inside the cylinder was washed two times with 250 μL of sterile H_2_O.

Bacteria were cultured according to the same protocol as described above and diluted to a concentration of 10^7^ CFU/mL. A volume of 100 μL of the *S. aureus* (ATCC 12600) suspension was applied to the wound area. The lid of the petri-dish was applied to create a moist chamber, and the skin was incubated for two hours at 37°C. A volume of 100 μL peptide solutions in H_2_O (0.1–2 mg/mL) or placebo (H_2_O) was added to the infected area and the sample was incubated for another four hours at 37°C. The liquid in the cylinder was removed and the remaining bacteria were harvested by adding 210 μL of Kligman buffer (0.1% Triton X-100 in 0.075 M phosphate buffer, pH 7.9) and the skin and wound inside the cylinder was scratched with a plastic loop using moderate force. The suspension was transferred to a micro-centrifuge tube, the procedure was repeated once and the two fractions of liquid from the infected area were pooled. The suspensions were diluted in four tenfold serial steps in diluted Kligman buffer (0.05% Triton X-100 in 0.0375 M phosphate buffer, pH 7.9) and an aliquot of 50 μL from each dilution was seeded on horse blood agar plates (Oxoid). The plates were incubated at 37°C for 24 h and plates containing 30–300 CFUs were counted. All personnel involved in the surgery, harvesting the bacteria and counting the plates were blinded to the different treatments.

### Statistical analysis

The data is presented as % of the control group ± SEM (%). The results were analysed with Student’s *t*-test, with a value of p < 0.05 considered statistically significant. Grubb’s test was performed to statistically identify outliers (Grubbs 1969).

## Results

### *In vitro* microbicidal effect of peptides derived from **centrocin 1**

An MMC_99_ assay was used to examine the ability of centrocin 1 heavy chain (CEN1 HC-Br), its debrominated analogue (CEN1 HC), and the C-terminal truncated variants of both peptides, *i.e.* CEN1 HC-Br (1–20) and CEN1 HC (1–20) to kill bacteria *in vitro*. In order to characterize the functional significance of a free cysteine residue in the sequence of centrocin 1 heavy chain, the cysteine to serine substituted variant CEN1 HC (Ser) was also included. The sequences of the peptides are shown in Table 1. The concentration that killed ≥99% bacteria was defined as MMC_99_. The experiments were performed using 0.037% BHI, which is the most commonly used media in this type of assessment. To mimic the physiological conditions in wounds, the data obtained in 0.037% BHI were complemented with experiments using 0.037% BHI supplemented with monovalent cations (85 mM or 150 mM NaCl), as well as using 50% of h.i. SWF.

All peptides evaluated exerted similar microbicidal activities in 0.037% BHI against *S. aureus* and *P. aeruginosa* (MMC_99_ = 12.5–25.0 mg/L; Table 2a and b). For CEN1 HC-Br and CEN1 HC, addition of monovalent cations (85 mM or 150 mM NaCl) to 0.037% BHI resulted in somewhat reduced microbicidal effect against *S. aureus* (MMC_99_ = 25.0–50.0 mg/L), while no reduction in effect was observed against *P. aeruginosa,* compared to 0.037% BHI only. For CEN1 HC-Br (1–20), CEN1 HC (1–20) and CEN1 HC (Ser), physiological-like sodium chloride concentrations completely abolished the bactericidal effect against *S. aureus* (MMC_99_ ≥ 200 mg/L) and reduced the effect against *P. aeruginosa* (MMC_99_ = 12.5–100.0 mg/L). When the MMC_99_ assay was performed in 50% h.i. SWF, the MMC_99_ values for CEN1 HC-Br and CEN1 HC were elevated to 25.0–50.0 mg/L for *S. aureus* and 100.0 mg/L for *P. aeruginosa*. No bactericidal effect was observed against *S. aureus* or *P. aeruginosa* with CEN1 HC-Br (1–20), CEN1 HC (1–20), and CEN1 HC (Ser) at concentrations up to 200 mg/L, when 50% h.i. SWF was used as assay medium (Table 2a and b).

**Table 2:** **Microbicidal effect of CEN1 HC-Br, CEN1 HC, CEN1 HC-Br (1–20), CEN1 HC (1–20) and CEN1 HC (Ser) against *S. aureus *(a) and *P. aeruginosa *(b)**

**(a)**				
	***S. aureus*, medium and MMC**_**99**_**(mg/L)**			
**Test substance**	**0.037% BHI**	**0.037% BHI + 85 mM NaCl**	**0.037% BHI + 150 mM NaCl**	**50% SWF**
CEN1 HC-Br	25	25	50	50
CEN1 HC	12.5	25	50	25
CEN1 HC-Br (1–20)	12.5	>200	>200	>200
CEN1 HC (1–20)	12.5	>200	>200	>200
CEN1 HC (Ser)	25	>200	>200	>200
**(b)**				
	***P. aeruginosa*, medium and MMC**_**99**_**(mg/L)**			
**Test substance**	**0.037% BHI**	**0.037% BHI + 85 mM NaCl**	**0.037% BHI + 150 mM NaCl**	**50% SWF**
CEN1 HC-Br	25	12.5	25	100
CEN1 HC	12.5	12.5	12.5	100
CEN1 HC-Br (1–20)	12.5	12.5	25	>200
CEN1 HC (1–20)	12.5	25	100	>200
CEN1 HC (Ser)	12.5	25	50	>200

The substances were tested using a MMC_99 _assay in concentrations up to 200 mg/L in four different assay media. Peptides were added to bacterial cells in duplicate (n = 2). Data are presented as maximum values from at least two independent experiments. h.i. = heat inactivated.

The microbicidal effect of CEN1 HC was further evaluated against a panel of Gram-positive bacteria (*S. aureus*, MRSA, *S. pyogenes, S. epidermidis*, and *P. acnes*) and Gram-negative bacteria (*E. coli, P. aeruginosa, K. pneumoniae,* and *A. baumannii*) as well as the yeast *C. albicans*. The results demonstrated that 3.1–6.3 mg/L or 6.3–100 mg/L of CEN1 HC was required to kill ≥99% of all the bacterial strains in 0.037% BHI broth and 50% h.i. SWF, respectively, indicating a broad range of microbicidal activities against both Gram-positive and Gram-negative bacteria assessed in both media. Interestingly, the ability to kill the yeast strain *C. albicans* appears to be impaired in 50% h.i. SWF (MMC_99_ ≥ 200 mg/L). The MMC_99_ values for CEN1 HC compared to the conventional antibiotics mupirocin (GlaxoSmithKline) and fusidic acid (LEO Pharma) are shown in Table 3.

**Table 3:** Microbicidal effect of CEN1 HC against Gram-positive and Gram-negative bacterial strains and a yeast strain

	**CEN1 HC MMC **_**99**_**(mg/L)**		**fusidic acid MMC**_**99 **_**(mg/L)**		**mupirocin MMC**_**99 **_**(mg/L)**	
**Microorganism, strain**	**0.037% BHI**	**50% h.i. SWF**	**0.037% BHI**	**50% h.i. SWF**	**0.037% BHI**	**50% h.i. SWF**
*S. aureus *ATCC 12600	6.3	12.5	6.3	6.3	3.1	3.1
MRSA ATCC 33591	6.3	25	6.3	6.3	3.1	3.1
*S. pyogenes *ATCC 12344	3.1	50	>200	>200	6.3	3.1
*S. epidermidis *ATCC 12228	3.1	12.5	6.3	6.3	3.1	3.1
*P. acnes *ATCC 6919	6.3	12.5	nd	nd	>200	>200
*E. coli *ATCC 11775	6.3	25	>200	>200	>200	>200
*P. aeruginosa *ATCC 15442	6.3	100	>200	>200	>200	>200
*K. pneumoniae *CCUG 59413	3.1	12.5	>200	>200	>200	>200
*A. baumannii *CCUG 58437	3.1	6.3	>200	>200	>200	>200
*C. albicans *ATCC 64549	6.3	>200	>200	>200	>200	>200

The substances were tested using a MMC_99 SS_assay in concentrations up to 200 mg/L in 0.037% BHI or 50% h.i. SWF. Peptides were added to bacterial cells in duplicate (n = 2). Data are presented as maximum values from at least two independent experiments. h.i. = heat inactivated, nd = not determined.

### *In vitro* anti-inflammatory effects of peptides derived from **centrocin 1**

**Figure 1:**
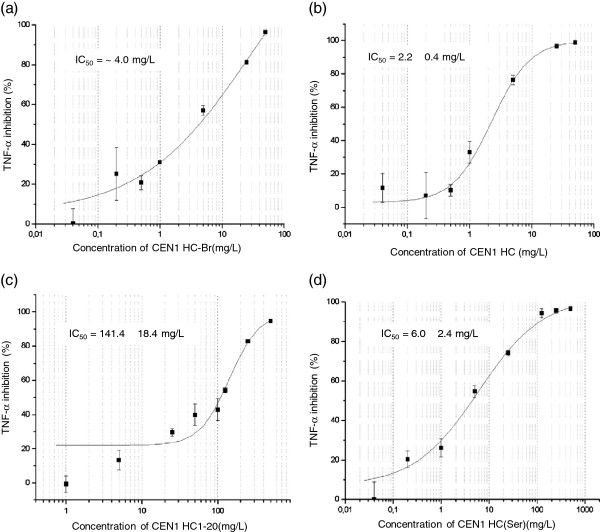
**Effects of CEN1 HC-Br (a), CEN1 HC (b), CEN1 HC (1–20) (c) and CEN1 HC (Ser) (d) on TNF-α secretion from THP-1 cells. **The peptides were added to PMA treated THP-1 cells in triplicate 30 min after the addition of LPS (0.1 ng/mL). Peptide concentrations ranging from 0.04 to 50 mg/L of CEN1 HC and CEN1 HC-Br, 0.04 to 500 mg/L of CEN1 HC (Ser) and 1 to 500 mg/L of CEN1 HC (1–20) were tested. Cytokine levels were measured in the cell supernatants by ELISA after six hours of stimulation. Data are presented as mean ± SEM with stimulated cytokine levels without peptide added set to 0%. Data was fitted to curves using a 4-parameter fit in the Origin software. The IC_50_ values were automatically calculated by the software. Due to an unsatisfactory curve fitting, IC_50_ for CEN1 HC-Br was estimated to be approximately 4 mg/L.

**Figure 2:**
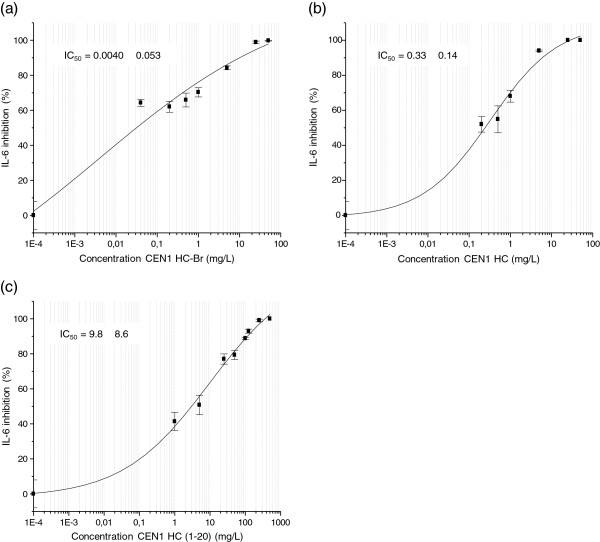
**Effects of CEN1 HC-Br (a), CEN1 HC (b) and CEN1 HC (1–20) (c) on IL-6 secretion from THP-1 cells. **The peptides were added to PMA treated THP-1 cells in triplicate 30 min after the addition of LPS (0.1 ng/mL). Peptide concentrations ranging from 0.04 to 50 mg/L of CEN1 HC and CEN1 HC-Br, and 1 to 500 mg/L CEN1 HC (1–20) were tested. Cytokine levels were measured in the cell supernatants by ELISA after six hours of stimulation. Data are presented as mean ± SEM with stimulated cytokine levels without peptide added set to 0%. Data was fitted to curves using a 4-parameter fit in the Origin software. The IC_50 _values were automatically calculated by the software.

The anti-inflammatory effect of CEN1 HC-Br, CEN1 HC, CEN1 HC (1–20) and CEN1 HC (Ser) was studied in macrophages derived from the human monocytic cell line THP-1. All peptides tested demonstrated a significant reduction in the release of the pro-inflammatory cytokine TNF-α in LPS-stimulated cells. However, the half maximal inhibitory concentration (IC_50_) value was markedly higher for the truncated variant CEN1 HC (1–20), compared to the other peptides (Figure 1a-d, Table 4). In line with these results, the peptides CEN1 HC-Br, CEN1 HC, CEN1 HC (1–20) reduced the production of another inflammation marker, IL-6, in LPS-stimulated cells, with the effect of CEN1 HC-Br and CEN1 HC being more pronounced compared to the truncated variant CEN1 HC (1–20) (Figure 2a-c, Table 4). In these experiments, the peptides were added to the cell culture medium 30 min after addition of LPS. Based on previous findings, this time interval should be sufficient for LPS to bind to the cell receptor and thus, to exclude that the peptide would neutralize the effect of LPS on cytokine production only by scavenging this agent (Elass-Rochard et al. 1998; Haversen et al. 2000). To rule out the possibility that the reduction in cytokine production by these peptides was related to peptide-induced decline in cell viability, the cell survival was measured after six hours of LPS-stimulation. The amount of viable cells after the treatment with CEN1 HC, CEN1 HC (1–20), and CEN1 HC (Ser) was approximately 90% of the cells treated with LPS only, indicating that addition of the peptides at concentrations up to 125 mg/L, did not show any significant cytotoxic effect. CEN1 HC-Br demonstrated higher cytotoxicity compared to the other peptides as the concentration of 100 mg/L reduced the amount of viable cells by approximately 50% (data not shown).

**Table 4:** Inhibitory effects of CEN1 HC-Br, CEN1 HC, CEN1 HC (1–20) and CEN1 HC (Ser) on TNF-α and IL-6 secretion from THP-1 cells

	**IC**_**50 **_**(mg/L)**	
**Test substance**	**TNF-α**	**IL-6**
CEN1 HC-Br	~4.0	0.0040 ± 0.053
CEN1 HC	2.2 ± 0.4	0.33 ± 0.14
CEN1 HC (1–20)	141.4 ± 18.4	9.8 ± 8.6
CEN1 HC (Ser)	6.0 ± 2.4	nd

Data are presented as half maximal inhibitory concentration (IC_50_) values ± SEM, as calculated by Origin software from dose–response curves using a 4-parameter fit. Due to an unsatisfactory curve fitting, IC_50_ for CEN1 HC-Br on TNF-α was estimated to approximately 4 mg/L. nd = not determined.

### *In vitro* resistance development towards CEN1 HC

The potential for resistance development against CEN1 HC was evaluated in two strains of Gram-positive bacteria, *S. aureus* and MRSA, by multistep dilution assay, and compared to the antibiotic mupirocin. For both strains the concentration of mupirocin killing ≥99% of the bacteria was significantly increased during 14 days of cultivation (from 3 to 24 mg/L for *S. aureus* and from 3 to >384 mg/L in MRSA). On the other hand, the concentration of CEN1 HC killing ≥99% of the bacteria was increased only twofold comparing values at day 0 and 14 (from 6 to 12 mg/L for both *S. aureus* and MRSA), which is within the limits of assay variation.

### Antimicrobial effect of CEN1 HC and CEN1 HC (Ser) in infected wound models

**Figure 3:**
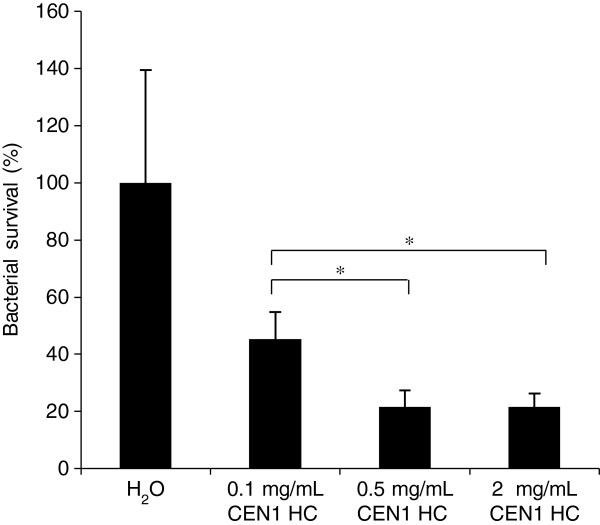
***In vivo *bacterial clearance of MRSA from excision wounds in rats after treatment with CEN1 HC. **Wounds infected with MRSA (4 × 10^7^ CFU) were treated with CEN1 HC, in concentrations of 0.1, 0.5, and 2 mg/mL, and sampling/bacterial counting was performed two hours later. Results are presented as mean relative bacterial survival (%) ± SEM compared to the non-treated control group (n = 14 for all the groups, except for the group treated with 0.5 mg/mL CEN1 HC, where n = 15). One value in each group, except for the group treated with 0.5 mg/mL of CEN1 HC, were considered outliers (p < 0.01, Grubbs’ test) and were discarded. * = p < 0.05.

**Figure 4:**
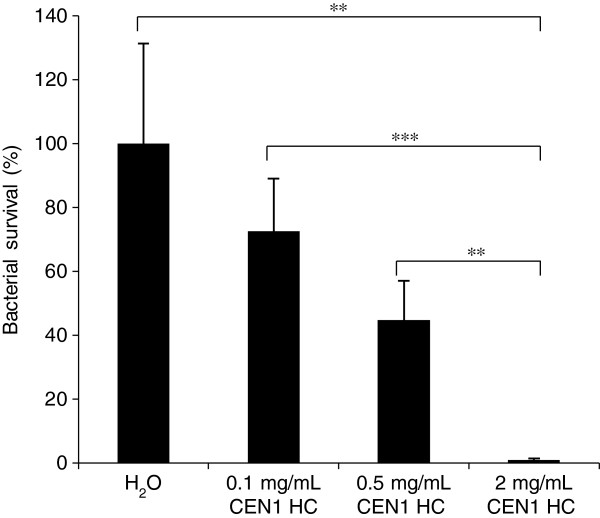
***In vivo *bacterial clearance of *P. aeruginosa *from excision wounds in rats after treatment with CEN1 HC. **Wounds infected with *P. aeruginosa *(4 × 10^7^ CFU) were treated with CEN1 HC, in concentrations of 0.1, 0.5, and 2 mg/mL, and sampling/bacterial counting was performed two hours later. Results are presented as mean of relative bacterial survival (%) ± SEM compared to the non-treated control group (n = 15 for all the groups). ** = p < 0.01, *** = p < 0.001.

In order to study a potential topical antimicrobial effect of CEN1 HC and CEN1 HC (Ser), an *in vivo* rat excision wound model and an *ex vivo* pig skin model were used. In the rat excision wound model full thickness wounds were infected with MRSA or *P. aeruginosa*, subsequently treated with peptide CEN1 HC or placebo (H_2_O), and bacterial counts were estimated two hours after the treatment. No animal death or local reactions in connection to the application of the test article were observed during the experimental period. CEN1 HC potently reduced the level of both bacterial strains in this model (Figures 3, 4). The effect of CEN1 HC (Ser) was evaluated only in wounds infected with MRSA, and this peptide failed to significantly reduce bacterial counts in this model (data not shown).

**Figure 5:**
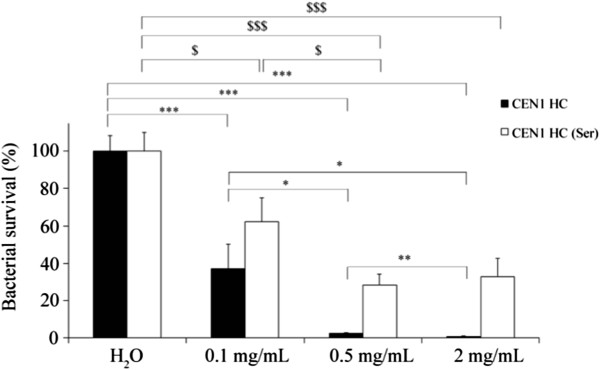
***Ex vivo *bacterial clearance of *S. aureus *from pig skin wounds after treatment with CEN1 HC and CEN1 HC (Ser).** Wounds infected with *S. aureus* (10^6^ CFU) were treated with CEN1 HC, in concentrations of 0.1, 0.5 and 2 mg/mL, and sampling/bacterial counting was performed four hours after treatment. Results are presented as mean of relative bacterial survival (%) ± SEM compared to the non-treated control group (n = 10 for all groups except for the groups treated with 0.5 and 2 mg/mL of CEN1 HC (Ser), where n = 9). One outlier (p < 0.01, Grubbs’ test) was discarded from each of the groups treated with 0.5 and 2 mg/mL CEN1 HC (Ser). * = p < 0.05, ** = p < 0.01, *** = p < 0.001 comparing CEN1 HC to the control group, $ = p < 0.05, $$$ = p < 0.001 comparing CEN1 HC (Ser) to the control group.

In the pig skin *ex vivo* study, punch biopsies were made on the skin from the back of pigs. The wounded areas were seeded with *S. aureus*, treated with CEN1 HC, CEN1 HC (Ser) or placebo (H_2_O), and bacterial counts were estimated four hours after treatment. As demonstrated in Figure 5, CEN1 HC reduced the level of bacteria in this model in a dose dependent manner. Treatment with CEN1 HC at the concentration of 0.5 and 2 mg/mL reduced bacterial counts with 98 and 99%, respectively, compared to the placebo treatment. Treatment with CEN1 HC (Ser) also significantly reduced the amount of bacteria. However, the effect was less pronounced compared to CEN1 HC (2 mg/mL of CEN1 HC (Ser) reduced bacterial counts with 67%, Figure 5).

## Discussion

The treatment of infectious diseases poses major challenges for healthcare systems worldwide due to rapid increase in bacterial strains resistant to conventional antibiotics. AMPs have emerged as novel promising agents for both topical and systemic treatment of infections. The AMPs are considered less prone to resistance development than conventional antibiotics due to the rapid microbicidal effect combined with broad mode of action (Fjell et al. 2012).

Drug development based on AMPs is complicated by the fact that the antimicrobial activities of numerous naturally occurring AMPs are antagonised by physiological salt concentrations, which might reflect the increasing ionic strength under high salt conditions weakening the electrostatic charge interactions of AMPs with bacterial membranes (Tam et al. 2002). Thus, attempts are directed towards developing therapeutic AMPs where salt-resistant properties are achieved through chemical modifications of the peptide structure (Fjell et al. 2012; Park et al. 2004; Tam et al. 2002). Peptides isolated from marine organisms, that are adapted to salt-rich environments, are likely to be less sensitive to ionic strength, and are therefore highly interesting from a drug development perspective.

In this study, the peptide previously isolated from coelomocyte (blood cell) extracts of the green sea urchin, *S. droebachiensis*, centrocin 1 heavy chain (CEN1 HC-Br) (Li et al. 2010) and the variants thereof, were evaluated for the potential as novel anti-infectious agents by measuring their antibacterial and anti-inflammatory effect *in vitro*. CEN1 HC-Br contains a brominated tryptophan in position two in the amino acid sequence (Li et al. 2010) with unknown function. AMPs containing brominated tryptophan have previously been characterized from several marine organisms such as hagfish intestinal antimicrobial peptides (HFIAPs), isolated from Atlantic hagfish or styelin D, isolated from tunicate *Styela clava* (Taylor et al. 2000; Uzzell et al. 2003). It has been speculated that the unusual amino acid bromotryptophan makes these AMPs less susceptible to proteolytic degradation and may increase the biological activity of the peptide (Li et al. 2010). To elucidate the functional significance of the bromination of the tryptophan residue, the debrominated variant of the centrocin 1 heavy chain, *i.e.* CEN1 HC, was included in the assessments. To map the peptide region necessary of the biological activity, the C-terminal truncated variants of both peptides, CEN1 HC-Br (1–20) and CEN1 HC (1–20), were also tested. A free cysteine residue in the peptide sequence may potentially complicate the product development process due to the possible heterogeneity of the product caused by formation of disulphide bonds between molecules. Therefore, we investigated if the cysteine residue in CEN1 HC (position 25) could be replaced by a similar amino acid. The most commonly used replacement for cysteine is serine, which in terms of geometry and volume occupancy is a highly isosteric analogue of cysteine. A cysteine to serine modified variant of CEN1 HC, CEN1 HC (Ser), was compared for microbicidal and anti-inflammatory properties to its non-substituted equivalent.

When the CEN1 HC-Br derived peptides were evaluated for anti-bacterial activity against *S. aureus* and *P. aeruginosa*, all peptides showed pronounced microbicidal effect in assay medium containing low salt concentrations and no serum (0.037% BHI). The peptides CEN1 HC-Br and CEN1 HC were generally salt and wound fluid tolerant and significant microbicidal activity was observed in the presence of 150 mM NaCl and in 50% h.i. SWF. Notably, physiological sodium chloride concentrations as well as h.i SWF severely decreased or fully eliminated the bactericidal effect of CEN1 HC-Br (1–20), CEN1 HC (1–20) and CEN1 HC (Ser). The microbicidal effect of the peptides and their sensitivity to physiological-like conditions varied dependently on the bacterial strain tested, possibly due to differences between the cell wall/membrane properties of the Gram-positive (*S. aureus*) and Gram-negative (*P. aeruginosa*) bacteria. Interestingly, debromination of tryptophan in CEN1 HC-Br did not impair the bactericidal effect in neither condition of low salt nor under physiological-like conditions.

The anti-inflammatory properties of CEN1 HC-Br, CEN1 HC, CEN1 HC (1–20) and CEN1 HC (Ser) were evaluated by measuring secretion of the most commonly used inflammation markers TNF-α and IL-6 in human monocyte derived macrophages stimulated with LPS. In this assay, CEN1 HC-Br, CEN1 HC and the Ser-substituted peptide CEN1 HC (Ser) exerted a more potent anti-inflammatory effect compared to the C-terminal truncated variant CEN1 HC (1–20). No obvious difference in efficacy was seen comparing CEN1 HC-Br and CEN1 HC, while the first peptide had higher cytotoxicity compared to the debrominated equivalent.

Based on these experiments, we conclude that debromination of the tryptophan residue of CEN1 HC-Br did not result in any reduced effect under the experimental conditions tested. Importantly, CEN1 HC had a more favourable safety profile compared to the original peptide, with no lytic activity observed against mammalian cells at the concentrations tested, thus showing a clear dissociation of antibacterial and anti-eukaryotic cell activities. C-terminal truncation of the full-length peptide variants resulted in reduction in salt-tolerability of the microbicidal effect as well as in reduced anti-inflammatory properties. Also, the peptide with the free cysteine residue, CEN1 HC had improved salt and serum tolerability profile in the MMC_99_ assay compared to the serine-modified equivalent. Based on these observations, it was concluded that from the panel of peptide variants tested, CEN1 HC showed the best efficacy and safety profile. Thus, CEN1 HC was selected to be further evaluated for its *in vitro* and *in vivo* antimicrobial properties.

CEN1 HC was shown to have broad microbicidal effect against microorganisms appearing in topical and parenteral infections including Gram-positive bacteria (*S. aureus*, *S. pyogenes, S. epidermidis,* and *P. acnes*), Gram-negative bacteria (*E. coli, P. aeruginosa, K. pneumonia,* and *A. baumannii*) and the yeast *C. albicans*. CEN1 HC also showed pronounced effect against the pathogen MRSA. Importantly, during the cultivation of 14 days, the bacterial strains tested (*S. aureus* and MRSA) failed to develop significant resistance towards CEN1 HC, indicating that these strains could not circumvent the action of this AMP, while significant resistance profile was observed toward the conventional antibiotic mupirocin used as comparator.

CEN1 HC demonstrated marked effect on reducing bacterial counts in infected wound models in two different animal species. In a rat model of infected full thickness excision wounds, treatment by CEN1 HC significantly reduced the bacterial counts of MRSA as well as *P. aeruginosa*, the most common pathogens in topical infections in man. The antibacterial effect against *S. aureus* was confirmed in an infected wound model in pig skin. In line with the *in vitro* tests, CEN1 HC (Ser) had markedly lower efficacy in these two animal models, compared to the cysteine-containing equivalent.

In summary, we demonstrate that CEN1 HC, a chemically synthesised 30 amino acid peptide sequentially derived from the previously described AMP centrocin 1, has a broad spectrum microbicidal effect, resistant to physiological salt concentration and serum containing wound fluid, combined with anti-inflammatory action. Importantly, *S. aureus* and MRSA failed to develop resistance against this peptide. CEN1 HC significantly reduced bacterial counts of *S. aureus*, MRSA and *P. aeruginosa* in animal models of infected wounds. Based on this study, CEN1 HC appears to be a promising agent in the topical treatment of infections, and further studies are warranted to evaluate the applicability of this AMP in clinical settings.

## Competing interests

Some of the authors of this paper have links to the Pergamum company. The links are as follows: Camilla Björn, Joakim Håkansson, Emma Myhrman, Veronika Sjöstrand, Kerstin Lindgren, and Margit Mahlapuu – fulltime employee at the time of the investigation. Pergamum contributed financially (including salaries), by providing laboratory space and payment for the study costs (animals and housing, drugs, and similar). All other authors declare that they have no competing interests.
